# Artificial Intelligence and Infectious Keratitis: Where Are We Now?

**DOI:** 10.3390/life13112117

**Published:** 2023-10-26

**Authors:** Mohammad Soleimani, Kasra Cheraqpour, Reza Sadeghi, Saharnaz Pezeshgi, Raghuram Koganti, Ali R. Djalilian

**Affiliations:** 1 Eye Research Center, Farabi Eye Hospital, Tehran University of Medical Sciences, Tehran 1336616351, Iran; msolei2@uic.edu (M.S.); cheraqpourk@gmail.com (K.C.); rsadeghi73@gmail.com (R.S.); 2 Department of Ophthalmology and Visual Sciences, University of Illinois at Chicago, Chicago, IL 60612, USA; rkogan3@uic.edu; 3 School of Medicine, Tehran University of Medical Sciences, Tehran 1461884513, Iran; saharnaz.pezeshki@gmail.com; 4 Cornea Service, Stem Cell Therapy and Corneal Tissue Engineering Laboratory, Illinois Eye and Ear Infirmary, Chicago, IL 60612, USA

**Keywords:** keratitis, infectious keratitis, microbial keratitis, artificial intelligence, AI, algorithm, diagnosis

## Abstract

Infectious keratitis (IK), which is one of the most common and catastrophic ophthalmic emergencies, accounts for the leading cause of corneal blindness worldwide. Different pathogens, including bacteria, viruses, fungi, and parasites, can cause IK. The diagnosis and etiology detection of IK pose specific challenges, and delayed or incorrect diagnosis can significantly worsen the outcome. Currently, this process is mainly performed based on slit–lamp findings, corneal smear and culture, tissue biopsy, PCR, and confocal microscopy. However, these diagnostic methods have their drawbacks, including experience dependency, tissue damage, cost, and time consumption. Diagnosis and etiology detection of IK can be especially challenging in rural areas or in countries with limited resources. In recent years, artificial intelligence (AI) has opened new windows in medical fields such as ophthalmology. An increasing number of studies have utilized AI in the diagnosis of anterior segment diseases such as IK. Several studies have demonstrated that AI algorithms can diagnose and detect the etiology of IK accurately and fast, which can be valuable, especially in remote areas and in countries with limited resources. Herein, we provided a comprehensive update on the utility of AI in IK.

## 1. Introduction

Infectious keratitis (IK) refers to a corneal infection. These infections can culminate in severe visual impairment or even permanent loss if not identified and treated in a timely manner. IK is the predominant etiology of corneal opacification and ranks fifth among the causes of blindness in developed and developing countries. It is responsible for 1.5–2.0 million new cases of monocular blindness annually worldwide [1]. The estimated incidence of infectious corneal blindness ranges from 2.5 to 799 per 100,000 population-years. In detail, 2.5–27.6, 2.6–40.3, and 6.6 per 100,000 population-years have been reported for the US, UK, and Australia, respectively, while undeveloped areas such as South India and Nepal experience rates of 113 and 799 per 100,000 population-years, respectively [2]. Contributing risk factors for IK include contact lens usage, ocular trauma, corneal diseases, lid abnormalities, and previous ocular surgeries [3]. In countries with advanced healthcare systems, there has been a recent increase in reported cases of IK due to the growing popularity of contact lenses. In the United States, the reported incidence rate is 2.5 per 100,000 individuals for non-contact lens wearers compared to 130.4 cases per 100,000 for contact lens wearers [4]. In contrast, underdeveloped countries have experienced persistent levels of IK nearly ten times as high as advanced nations such as the United States [4].

**Figure 1:**
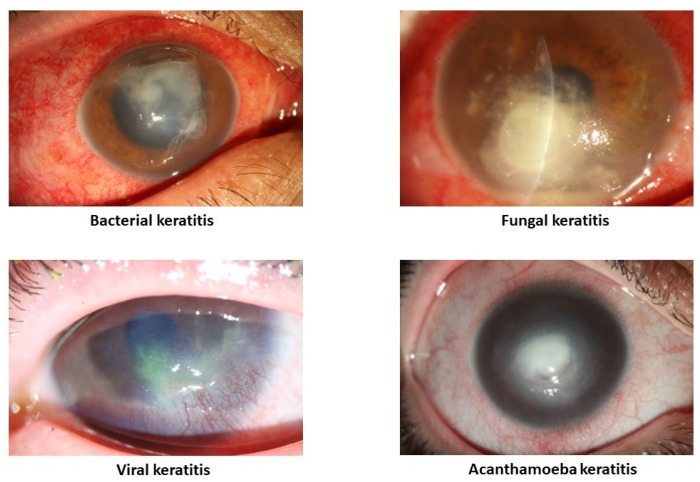
Different subtypes of infectious keratitis.

IK can be caused by a multitude of agents, including bacteria, viruses, fungi, and parasites (Figure 1). The primary characteristic of IK is the presence of a corneal infiltrate, an area of white/yellow corneal haze or opacity with a layer of damaged epithelium [5]. However, these characteristics may vary between different etiologies and, notably, different microorganisms may occasionally manifest with specific presentations. Currently, the diagnosis of IK and subsequent identification of etiology are mainly made based on slit–lamp findings, corneal smear and culture, tissue biopsy, PCR, and confocal microscopy. However, these diagnostic methods have their drawbacks, including experience dependency, tissue damage, cost, and time consumption. This process can be especially challenging in rural areas or in countries with limited resources.

A subfield of computer science known as artificial intelligence (AI) consists of research and development of new technical fields that simulate and advance human intelligence theory, methods, and application systems [6]. AI subclassifications include machine learning (ML), deep learning (DL), artificial neural networks (ANNs), deep neural networks (DNNs), convolution neural networks (CNNs), and transfer learning [7]. The utilization of AI can significantly benefit society and the healthcare system, given that the diagnosis of ocular conditions heavily relies on the experience of highly trained ophthalmologists whose distribution varies significantly across geographic areas [8,9]. Additionally, given the widespread use of imaging tools in clinical practice and the consequent availability of codified data from imaging to numeric clinical parameters, the ophthalmic community is positioned well to develop AI strategies [10]. The first ophthalmic AI device to automatically diagnose and grade diabetic retinopathy, IDx-DR, was approved by the U.S. Food and Drug Administration in 2018 [11]. Previously, ocular AI research was mainly focused on diseases of the posterior segment, such as diabetic retinopathy, retinopathy of prematurity, age-related macular degeneration, retinal vein occlusion, and glaucoma optic neuropathy [11,12,13,14,15]. However, an increasing number of studies have utilized AI in the diagnosis of anterior segment diseases such as IK [16,17,18]. Incorporating AI into the diagnosis, etiology detection, and management of IK can provide a cutting-edge solution to the shortage of ophthalmologists and improve patient care and outcomes. AI algorithms can be trained to recognize patterns in images of the eye that are invisible to the naked eye, which allows AI algorithms to diagnose IK and determine etiology with a high degree of accuracy. Several studies have demonstrated that AI algorithms can diagnose IK from slit–lamp images with similar accuracy as human experts [19]. AI algorithms have also been trained to diagnose fungal keratitis from confocal microscopy images with satisfactory diagnostic performance [20]. These studies suggest that AI has the potential to be a valuable tool for the diagnosis of IK and etiology detection, especially in remote areas and in countries with limited resources. Herein, we provide a comprehensive update on the utility of AI in IK. Since this technology can be utilized in numerous ways, we have classified the models into diagnostic and discriminative models, which target primary healthcare practitioners and specialists such as ophthalmologists, respectively.

## 2. Methodology

The English-language literature published from January 2002 to June 2023 on PubMed/Medline, ISI Web of Knowledge, ScienceDirect, Scopus, and Google Scholar databases was searched using the following keywords: “keratitis” OR “infectious keratitis” OR “corneal infection” OR “corneal ulcer” AND “artificial intelligence”, OR “AI” OR “deep learning”, OR “machine learning”, OR “neural network”. The extracted articles were first reviewed by title and abstract, followed by the examination of full texts of eligible articles and their corresponding reference list. Non-English articles, preprints, and computational simulations were excluded.

## 3. Historical Point of View

Historically, the first attempted application of AI in the diagnosis of IK utilized an artificial neural network (ANN), which incorporated three categories of information as inputs: (1) ocular predisposing factors (such as trauma, contact lens wear, lid and lacrimal diseases, and steroid usage), (2) systemic risk factors (such as diabetes, alcoholism, and immune deficiency disorders), and (3) ulcer features (such as size, depth, and location) [21]. In this study by Saini et al. in 2003, the trained model could correctly classify all sixty-three corneal ulcers in the training set. In the test set, their model correctly classified 39 out of 43 corneal ulcers. Specificity for bacterial and fungal categories was 76.47% and 100%, respectively. Additionally, the accuracy of classification was 90.7%, which was significantly better than the clinicians’ average accuracy of 62.8%. Although imaging was not used in this study, the trained model demonstrated significant potential in accurately classifying of corneal ulcers based on forty input variables from each case. In later studies, AI was used to process and classify images. Initially, slit–lamp or other imaging modalities were used, and AI could only confirm or rule out keratitis. However, this was still a valuable tool for quick diagnosis with a comparable accuracy to ophthalmologists. Later iterations incorporated the ability to image corneal layers in 3D using a confocal microscope. Currently, most projects are using advanced AI models to not only diagnose but even discriminate between types of IK.

## 4. Slit–Lamp/External Photography-Based Studies

### 4.1. Diagnostic Models

As technology progressed, AI gradually began interpreting images in detail. Loo et al. developed a fully automatic algorithm for the segmentation of ocular structures and biomarkers of IK on slit–lamp photographs under two different illuminations [22]. Their algorithm was able to identify stromal infiltration, hypopyon, WBCs, and edema on diffuse white-light images and epithelial defects, corneal limbus, and light reflexes on diffuse blue-light images with fluorescein staining. The Dice similarity coefficient (DSC) of the algorithm compared to a physician in this study was 0.95, a highly favorable result for AI. The researchers made their datasets and algorithms freely available as an open-source software package to promote the future development of automatic algorithms for determining the diagnosis and prognosis of infectious diseases of the cornea.

In one study on fungal keratitis, Li et al. assessed the impact of the segmentation method (e.g., manual or automated) on diagnostic accuracy and speed [23]. They hypothesized that combining manual and automated segmentation methods may yield greater efficiency. They found that automated segmentation is more accurate than manual segmentation. However, manual segmentation shows faster performance compared to automated segmentation.

In an interesting multi-center report, the differentiation between active corneal ulcers and healed scars was studied [24]. The trained model was tested on two different patient populations from eye clinics in India (n = 200) and the Byers Eye Institute at Stanford University (n = 101). The results were promising: 115/123 active ulcers and 65/77 scars from the Indian population and 43/55 active ulcers and 42/46 scars from the Northern California population were correctly diagnosed.

Kuo et al. [25] developed a deep learning model to diagnose fungal keratitis cases from corneal photography. The results were compared with opinions of three experienced cornea specialists (with at least 7 years of qualification in the specialty) and three non-cornea specialty ophthalmologists with comparable qualifications in clinical practice. Their model showed an average of around 70% accuracy, which was higher than the non-cornea specialty ophthalmologists but lower than the cornea specialists. Hence, such models can be logically applied in rural areas where highly specialized medical personnel are not readily available.

Comparing the performance of different deep learning models in the detection of bacterial keratitis from corneal photographs was the subject of another study by Kuo et al. [26]. EfficientNet and non-EfficientNet models were assessed in this study. The results showed that all models have a comparable accuracy. However, non-EfficientNet models showed a higher sensitivity (79–82% and 50–55% of sensitivity and specificity, respectively), and EfficientNet models had a higher specificity (73–74% and 60–64% of sensitivity and specificity, respectively). Overall, all deep learning models had a higher sensitivity but lower specificity in the diagnosis of bacterial keratitis compared to ophthalmologists.

Wei et al. [27] conducted a large study regarding the diagnosis of fungal keratitis. They mixed manual recognition and machine learning systems in a multi-center study with promising results.

Natarajan and colleagues [28] conducted one of the limited number of studies regarding viral keratitis. A total of 307 slit–lamp images from 285 eyes were included. Out of these, 177 images were from patients with necrotizing HSV keratitis, and the remaining 130 images belonged to patients with bacterial and fungal keratitis. A rate of 72%, 69.6%, and 76.5% were reported for the accuracy, sensitivity, and specificity of the model, respectively.

Alongside qualitative analysis, AI seems to have the potential to provide quantitative analytics. Although diagnosis and classification are the common subjects of AI studies in the field of IK, assessing the severity of the disease can be another interesting target. In line with this concept, Alquran et al. [29] used a model to evaluate various parameters, including ulcer pattern (point-like, point-flaky, flaky), type (no ulcer, micro punctate, macro punctate, coalescent macro punctate, and patch ≥ 1 mm), and grade (from grade 0 or no ulcer to grade 4 or central optical zone involvement) in fluorescein staining images.

### 4.2. Discriminative Models

As AI algorithms and diagnostic efficacy began improving, new studies were performed to identify whether the models could distinguish between the various microbial etiologies of IK. Fungal keratitis is notoriously difficult to distinguish from bacterial keratitis in clinical practice. In a study by Hung and colleagues [30], a deep learning algorithm was applied to differentiate between slit–lamp photos of bacterial and fungal keratitis. They achieved an average accuracy of approximately 80%, surpassing general ophthalmologists and comparable to cornea specialists. In detail, the accuracy rate for bacterial and fungal keratitis ranged from 79.6% to 95.9% and 26.3% to 65.8%, respectively.

Another study by Ghosh et al. [31] aimed to differentiate between fungal and bacterial keratitis as well, and their algorithm showed an accuracy of 83% in this regard. They found that image brightness plays a significant role in the correct classification of fungal keratitis.

One study by Won et al. [32] developed a deep learning algorithm that was strengthened with two modules in order to discriminate between bacterial and fungal keratitis. Notably, adding the two mentioned modules could significantly increase the accuracy of the algorithm from 81.1% to 87.8%.

Combining different types of AI models may improve diagnostic performance. In a large study by Zhang and colleagues [33], 1490, 1670, 600, and 1070 patients with bacterial, fungal, herpes simplex virus (HSV), and Acanthamoeba keratitis were enrolled, respectively. Different combinations of models were tested, and the highest accuracy was 77.08%. Bacterial, fungal, Acanthamoeba, and HSV keratitis were diagnosed with an accuracy of 70.27%, 77.71%, 83.81%, and 79.31%, respectively. Interestingly, AI’s accuracy for each subtype of keratitis exceeded that of three invited cornea specialists. In contrast, Kuo et al. [34] stated that although ensemble models can enhance performance, the effect seems to be limited. They introduced a deep learning model to distinguish Pseudomonas keratitis from non-Pseudomonas bacterial keratitis. Various algorithms were used as the backbone, and 71.2% was the highest reported accuracy. However, the best ensemble model showed an accuracy of 72.1%, which was not a statistically significant difference.

Moreover, in a study by Hu and colleagues [35], six deep learning algorithms were tested and compared with the performance of two ophthalmologists. Out of these, the best algorithm showed an accuracy and a specificity of 73.5% and 90.4%, respectively, in differentiating between different keratitis types.

Koyama et al. [36] developed a hybrid deep learning algorithm to identify the causative pathogen from slit–lamp images. One of the novel aspects of their work was applying facial recognition techniques to provide the ability to analyze images from different angles and with different levels of illumination and degrees of resolution. The overall accuracy rate of the multiclass diagnosis was 88.0%. In detail, Acanthamoeba, bacteria, fungi, and HSV were accurately diagnosed with rates of 97.9%, 90.7%, 95.0%, and 92.3%, respectively. The algorithm showed a significantly higher performance than 35 invited board-certified ophthalmologists throughout Japan, including 16 cornea specialist faculty members.

In another study, Sajeev et al. [37] developed a deep learning approach to differentiate between bacterial and viral keratitis, which showed an acceptable performance. Also, Wang et al. [38] developed a deep learning-based model to differentiate between bacterial, fungal, and HSV keratitis. Notably, they used photographs acquired by slit–lamp and smartphones. Promising results were reported for their model.

Redd and colleagues [39] developed models for automated image-based differentiation of bacterial and fungal ulcers using images from handheld portable cameras. They proposed that telemedicine applications using less-expensive and more portable imaging methods may significantly increase the potential public health impact of this technology since low-income countries may have limited potential for implementation of models that require slit–lamp mounted cameras. In their multi-center study, different algorithms were used to discriminate between fungal and bacterial ulcers, and the results were compared to human experts. Interestingly, their models achieved superhuman performance. Similarly, Xu et al. [40] designed a model that could greatly exceed the average level of professionals and reach the level of performance of top ophthalmologists.

Table 1 provides a summary of different slit–lamp photography-based studies.

## 5. Confocal Microscopy-Based Studies

One of the most useful imaging modalities in the diagnosis of infectious keratitis, particularly for fungi and Acanthamoeba, is in vivo confocal microscopy (IVCM). IVCM is considered a non-invasive technique that can provide high-resolution images at cellular and sub-cellular levels. Its utility is especially prominent in microbiological negative cases or deep infiltration, which blocks access from obtaining tissue for smear and culture [43].

### 5.1. Diagnostic Models

AI systems gradually became more complex and were trained to diagnose subtypes of keratitis utilizing confocal imaging. For example, in the diagnosis of fungal keratitis, distinguishing between nerves of a normal cornea and fungal hyphae on confocal microscopy images is a remarkable challenge, especially with a complicated background. To solve this issue, Wu et al. [44] introduced a novel automatic hyphae detection method to classify the normal and abnormal images. Hyphal density was then calculated using the ratio of hyphae length to the area as a marker for the infection severity. This method resulted in an accuracy of nearly 100% in distinguishing between normal and abnormal images.

In another successful application of AI to confocal imaging [20], a total of 535 images were tested, which showed a satisfactory performance of 515 correct diagnoses and 20 misdiagnoses (6 with fungal hyphae and 14 without).

In a hallmark study for the accurate diagnosis of fungal keratitis by Liu and colleagues [45], a novel convolutional neural network framework for the diagnosis of fungal keratitis from confocal microscopy images was introduced. They compared the accuracy of two traditional models with similar models based on mean fusion and histogram-matching fusion. Their results showed that using data augmentation and image fusion has a diagnostic accuracy of 99.95%.

Hou et al. [46] proposed a web-based medical image management and analysis system. Then, they integrated their model with a system designed to diagnose confocal microscopy images of fungal keratitis online.

### 5.2. Discriminative Models

In a study by Zhang and colleagues [47], they classified bacterial, fungal, and viral keratitis based on confocal microscopy images. Their results showed 75% and 70.13% of average accuracy and sensitivity, respectively. However, the sensitivity for diagnosing viral keratitis was comparatively lacking at only 16.8%.

In another study by Tang et al. [48], confocal microscopy images were used to identify pathogenic fungal genera. The results were satisfactory regarding the identification of Fusarium and Aspergillus, with an accuracy rate of 81.7% and 75.7% for the detection of the two fungi, respectively.

In another study, Essalat et al. [49] enrolled confocal microscopy images of fungal, Acanthamoeba, non-specific keratitis, and normal corneas. Their study reported sensitivity and specificity of 91.4% and 98.3% for Acanthamoeba and 97.0% and 96.4% for fungal keratitis, respectively. Notably, the authors have presented their confocal microscopy-keratitis dataset openly available for future research. Table 2 provides a summary of different confocal microscopy-based studies.

## 6. What about Culture-Negative Ulcers?

In a study by Kogachi and colleagues [42], different algorithms were trained to determine whether CNNs are capable of differentiating morphological differences of slit–lamp photographs between images of culture-positive and -negative corneal ulcers. Notably, their models could not detect morphological differences between microbiologically positive and negative corneal ulcers. Hence, the authors concluded that although current models have used only microbiologically positive cases, the results are potentially generalizable even to cases with negative microbiological results [42]. However, more studies are required to address this issue.

## 7. Challenges and Limitations

Some technical challenges still exist which impede the accurate diagnosis of IK and should be addressed in future studies. First, the current models are greatly dependent on image quality. Most studies have used high-quality images, but capturing high-quality images is not possible in all medical centers, and transferring the images via messaging platforms may decrease quality as well. However, there are successful models with image quality around 200 × 200 to 300 × 300 pixels. Other image-related factors which may impact diagnostic accuracy include brightness, orientation, and focus. A standard range has not yet been defined for these parameters. Hence, it can be expected that the reported performances may fluctuate in different settings. Second, pre-existing ocular pathologies such as pterygium, corneal arcus, conjuctivalization, and surgical scars can severely impact the results. Third, although mixed infections (e.g., two or more types of pathogens) are not uncommon in routine practice, none of the previous studies have studied polymicrobial variants of IK. Moreover, the depth of corneal involvement can act as a differentiating factor for etiological detection, as well as a prognostic and severity parameter, which can predict subsequent complications such as endophthalmitis. This factor is routinely noted during routine practice using slit–beam illumination, but current models mainly use diffuse-beam photos. Therefore, the depth of involvement remains unconsidered. More advanced algorithms with larger sample sizes can overcome these challenges.

Collectively, it seems that although the current models have shown promising results, a long way is still to go in applying these models in routine practice. In fact, in clinical practice, the scenarios that ophthalmologists face are more complex; patients with pre-existing pathologies, previous ocular surgeries, and mixed or negative culture cases are among these conditions, which the current models are not trained to deal with. However, as stated earlier, these technologies can be applied at different levels. So, diagnostic models, especially those enrolled in other ocular surface pathologies in their dataset, can be helpful in the screening or primary healthcare level. Hence, we should emphasize that clinical diagnosis remains a critical initial step in managing infectious keratitis, and artificial intelligence-based models would never substitute for the human brain and decision making, and their role remains at the level of auxiliary tools.

## 8. Future Directions

The field of AI is rapidly evolving, and multiple novel applications for AI in IK are being developed. As AI technology continues to progress, it is likely to play an increasingly important role in the diagnosis and management of IK. They can help to improve access to eye care and reduce the risk of blindness. Hence, besides diagnosis alone, future studies are required to focus on identifying the severity of the disease, prognosis, probability of recurrence, and treatment approach and regimen. On a related note, AI can be used to screen databases for new therapeutic treatments for IK as well. We believe that, in the near future, more advancements can revolutionize this field, such as AI-powered devices like microscopes to automatically detect and classify microorganisms in corneal smears and culture, AI-powered telemedicine assistants to provide remote consultations for patients in rural areas and in countries with limited resources, and AI-powered decision support systems to help ophthalmologists diagnose and treat IK, improving the quality of care and reducing the risk of complications from human error.

## 9. Conclusions

Infectious keratitis is a serious eye infection that can lead to blindness if not treated promptly. The diagnosis and etiology detection of infectious keratitis pose specific challenges, and delayed or incorrect diagnosis can significantly worsen outcomes. AI is a promising new technology that has the potential to revolutionize the diagnosis and treatment of infectious keratitis. AI algorithms can be used to diagnose and identify the etiology of infectious keratitis with a high degree of accuracy, even in remote areas and in countries with limited resources. Other potential roles of AI include developing new treatments, monitoring the progression of the disease, and personalizing the treatment for each patient. As AI technology continues to develop, it is likely to play an increasingly important role in the fight to preserve eyesight against infectious keratitis.

## Figures and Tables

**Table 1:** A brief summary of slit–lamp/external photography-based studies.

Publication Year	First Author	Study Model	Sample Size (Image)	Image Acquisition	Keratitis Type	AI Algorithm	AUC (%)	Acc. (%)	Sen. (%)	Spe. (%)
2020	Kuo [25]	Diagnostic	288	Nikon (Tokyo, Japan) D100 camera/Canon (Tokyo, Japan) EOS 7D camera	FK	DenseNet	65.00	69.40	71.10	68.40
2021	Kuo [26]	Diagnostic	1512	Resolution of 224 × 224 pixels	BK	ResNet, DenseNet, EfcientNet	80.00	70.30	74.10	64.30
2021	Tiwari [24]	Diagnostic	2445	Handheld Nikon D-series digital SLR camera with a 105 mm f/2.8D AF Micro Nikkor Autofocus Lens and a modified Nikon SB29s electronic flash or Nikon R1 Wireless Close-up Speedlight system/Canon 7D digital camera, Haag-Streit BX900	FK, BK	VGG-16	97.31	-	93.50	84.42
2021	Li [41]	Diagnostic	13,557	Resolution of 224 × 224 pixels	FK, BK, VK	DenseNet121, inception-v3, ResNet50	99.80	98.00	97.70	98.20
2022	Alquran [29]	Diagnostic	712	Resolution of 256 × 256 pixels	Ulcer	ResNet101	-	93.90	93.32	-
2023	Li [23]	Diagnostic	423	Slit photography/super macro mode of HUAWEI (Shenzhen, China) P30	FK	DL, LASSO, MLP	83.90	77.65	86.05	76.19
						DL, LASSO, MLP + automatic segmentation program	92.50	84.52	90.48	85.71
2023	Wei [27]	Diagnostic	420	-	FK	Binary logistic regression, Randomforest classification, Decision tree classification	90.30	90.50	90.70	89.90
2023	Natarajan [28]	Diagnostic	307	8.1 MP digital camera via a DC-3 digital camera/resolution of 224 × 224 pixels	VK	DenseNet	73.00	72.00	69.60	76.50
2021	Xu [40]	Discriminative	2284	SL Cam/digital camera Unit DC-1	BK, FK, HK	VGG-16, GoogLeNet-v3, DenseNet	80.00	-	-	-
2021	Wang [38]	Discriminative	6073	Nikon DSC D5200 camera/SANYO VPC-MZ3GX camera/smartphone/resolution of 299 × 299 pixels	BK, FK, HK	InceptionV3, ResNet50, DenseNet121	95.07	-	-	-
2021	Koyama [36]	Discriminative	4036	EOS kiss × 7 (Canon)/α6000 (Sony, Tokyo, Japan)/D90 (Nikon)	FK	ResNet-50, InceptionResNetV2	97.50	95.00	-	-
					BK		96.30	90.70	-	-
					AK		99.50	97.90	-	-
					HK		94.60	92.30	-	-
2021	Hung [30]	Discriminative	1330	Canon EOS 7D camera/Nikon D100 camera/Canon EOS 7D camera	FK, BK	CNN	85.00	78.60	65.80	87.30
2021	Sajeev [37]	Discriminative	446	Resolution of 256 × 256 pixels	BK, VK	CNN	85.60	81.20	71.50	88.00
2022	Zhang [33]	Discriminative	4830	Resolution of 224 × 224 pixels	AK	ResNext101_32x16d + DenseNet169	96.00	83.81	78.00	-
					HK		98.00	79.31	80.00	-
					BK		86.00	70.27	70.00	-
					FK		91.00	77.71	78.00	-
2022	Ghosh [31]	Discriminative	2167	Canon EOS 7D camera/resolution of 256 × 256 pixels	FK, BK	VGG19, ResNet-50, DenseNet-121	90.40	83.00	77.00	-
2022	Kuo [34]	Discriminative	929	Resolution of 224 × 224 pixels	BK	ResNet50, DenseNet121, ResNeXt50, SE-ResNet50	82.00	72.10	79.60	57.2
2022	Redd [39]	Discriminative	980	Handheld Nikon D-series digital single-lens reflex camera	FK, BK	MobileNetV2, DenseNet201, ResNet152V2, VGG19, Xception	86.00	-	-	-
2023	Kogachi [42]	Discriminative	1970	Handheld Nikon D7100 camera with a 105 mm macro lens/resolution of 224 × 224 pixels	FK, BK	DenseNet, MobileNet	56.00	NR	NR	NR
2023	Hu [35]	Discriminative	2757	DC-4 digital cameras	FK, BK, VK	EffecientNetV2-M	85.00	73.50	68.00	90.40
2023	Won [32]	Discriminative	684	Resolution of 500 pixels × 750 pixels	FK, BK	ResNet-50 + LGM & MAM	89.00	87.80	86.40	88.70

AI, artificial intelligence; AK, Acanthamoeba keratitis; BK, bacterial keratitis; CNN, convolutional neural networks; DL, deep learning; FK, fungal keratitis; HK, herpes simplex keratitis; LASSO, least absolute shrinkage and selection operator; LGM, lesion guiding module; MAM, mask-adjusting module; MLP, multilayer perception; VGG, visual geometry group; VK, viral keratitis; AUC, area under the curve; Acc, accuracy; Sen, sensitivity; Spe, specificity.

**Table 2:** A brief summary of confocal microscopy-based studies.

Year	First Author	Study Model	Sample Size (Image)	Keratitis	AI Algorithm	AUC (%)	Acc. (%)	Sen. (%)	Spe. (%)
				Type					
2018	Wu [44]	Diagnostic	378	FK	ARBP + SVM	99.01	99.74	100	99.45
2019	Liu [45]	Diagnostic	1213	FK	DCNN/HMF	-	99.95	99.9	100
2020	Lv [20]	Diagnostic	2088	FK	ResNet	97.69	93.64	82.56	98.89
2021	Hou [46]	Diagnostic	1870	FK	AlexNet, ZFNet, VGG16	99.97	99.29	99.33	99.62
2020	Zhang [47]	Discriminative	3321	VK	ResNet	-	99.09	16.8	-
				BK	ResNet	-	80	89.72	-
				FK	ResNet	-	96.36	71.71	-
2022	Tang [48]	Discriminative	3364	*Fusarium*	DL/ADT	88.7	81.7	79.1	83.1
				*Aspergillus*		82.7	75.7	75.6	75.9
2023	Essalat [49]	Discriminative	4001	AK	CNN	-	95.7	91.37	98.25
				FK		-	96.5	96.98	96.38

ADT, abstract data type; AI, artificial intelligence; AK, Acanthamoeba keratitis; ARBP, adaptive robust binary pattern; AUC, area under the curve; BK, bacterial keratitis; CNN, convolutional neural networks; DCNN, deep convolutional neural networks; DL, deep learning; FK, fungal keratitis; HMF, histogram matching fusion; SVM, support vector machine; VK, viral keratitis; AUC, area under the curve; Acc, accuracy; Sen, sensitivity; Spe, specificity.

## Data Availability

Not applicable.

## References

[1] Shah S., Rana D., Purohit D., Malhotra S. (2021). Evaluation Of Therapeutic Modalities And Outcome In Patients Of Infectious Keratitis: A Prospective Observational Study At A Tertiary Care Eye Hospital. Natl. J. Integr. Res. Med..

[2] Ting D.S.J., Ho C.S., Deshmukh R., Said D.G., Dua H.S. (2021). Infectious keratitis: An update on epidemiology, causative microorganisms, risk factors, and antimicrobial resistance. Eye.

[3] Soleimani M., Tabatabaei S.A., Masoumi A., Mirshahi R., Ghahvechian H., Tayebi F., Momenaei B., Mahdizad Z., Mohammadi S.S. (2021). Infectious keratitis: Trends in microbiological and antibiotic sensitivity patterns. Eye.

[4] Egrilmez S., Yildirim-Theveny Ş. (2020). Treatment-resistant bacterial keratitis: Challenges and solutions. Clin. Ophthalmol..

[5] Dalmon C., Porco T.C., Lietman T.M., Prajna N.V., Prajna L., Das M.R., Kumar J.A., Mascarenhas J., Margolis T.P., Whitcher J.P. (2012). The clinical differentiation of bacterial and fungal keratitis: A photographic survey. Investig. Ophthalmol. Vis. Sci..

[6] Sha W., Guo Y., Yuan Q., Tang S., Zhang X., Lu S., Guo X., Cao Y.-C., Cheng S. (2020). Artificial intelligence to power the future of materials science and engineering. Adv. Intell. Syst..

[7] Rampat R., Deshmukh R., Chen X., Ting D.S., Said D.G., Dua H.S., Ting D.S. (2021). Artificial intelligence in cornea, refractive surgery, and cataract: Basic principles, clinical applications, and future directions. Asia-Pacific J. Ophthalmol..

[8] Resnikoff S., Lansingh V.C., Washburn L., Felch W., Gauthier T.-M., Taylor H.R., Eckert K., Parke D., Wiedemann P. (2020). Estimated number of ophthalmologists worldwide (International Council of Ophthalmology update): Will we meet the needs?. Br. J. Ophthalmol..

[9] Ledbetter E.C. (2022). Applications of in vivo confocal microscopy in the management of infectious keratitis in veterinary ophthalmology. Vet. Ophthalmol..

[10] Chia M.A., Turner A.W. (2022). Benefits of integrating telemedicine and artificial intelligence into outreach eye care: Stepwise approach and future directions. Front. Med..

[11] Grzybowski A., Brona P., Lim G., Ruamviboonsuk P., Tan G.S., Abramoff M., Ting D.S. (2020). Artificial intelligence for diabetic retinopathy screening: A review. Eye.

[12] Campbell J.P., Chiang M.F., Chen J.S., Moshfeghi D.M., Nudleman E., Ruambivoonsuk P., Cherwek H., Cheung C.Y., Singh P., Kalpathy-Cramer J. (2022). Artificial intelligence for retinopathy of prematurity: Validation of a vascular severity scale against international expert diagnosis. Ophthalmology.

[13] Romond K., Alam M., Kravets S., Sisternes L.d., Leng T., Lim J.I., Rubin D., Hallak J.A. (2021). Imaging and artificial intelligence for progression of age-related macular degeneration. Exp. Biol. Med..

[14] Chen Q., Yu W.-H., Lin S., Liu B.-S., Wang Y., Wei Q.-J., He X.-X., Ding F., Yang G., Chen Y.-X. (2021). Artificial intelligence can assist with diagnosing retinal vein occlusion. Int. J. Ophthalmol..

[15] Li F., Su Y., Lin F., Li Z., Song Y., Nie S., Xu J., Chen L., Chen S., Li H. (2022). A deep-learning system predicts glaucoma incidence and progression using retinal photographs. J. Clin. Investig..

[16] Ting D.S.J., Foo V.H., Yang L.W.Y., Sia J.T., Ang M., Lin H., Chodosh J., Mehta J.S., Ting D.S.W. (2021). Artificial intelligence for anterior segment diseases: Emerging applications in ophthalmology. Br. J. Ophthalmol..

[17] Zhang Z., Wang Y., Zhang H., Samusak A., Rao H., Xiao C., Abula M., Cao Q., Dai Q. (2023). Artificial intelligence-assisted diagnosis of ocular surface diseases. Front. Cell Dev. Biol..

[18] Ji Y., Liu S., Hong X., Lu Y., Wu X., Li K., Li K., Liu Y. (2022). Advances in artificial intelligence applications for ocular surface diseases diagnosis. Front. Cell Dev. Biol..

[19] Vupparaboina K.K., Vedula S.N., Aithu S., Bashar S.B., Challa K., Loomba A., Taneja M., Channapayya S., Richhariya A. (2019). Artificial intelligence based detection of infectious keratitis using slit-lamp images. Investig. Ophthalmol. Vis. Sci..

[20] Lv J., Zhang K., Chen Q., Chen Q., Huang W., Cui L., Li M., Li J., Chen L., Shen C. (2020). Deep learning-based automated diagnosis of fungal keratitis with in vivo confocal microscopy images. Ann. Transl. Med..

[21] Saini J.S., Jain A.K., Kumar S., Vikal S., Pankaj S., Singh S. (2003). Neural network approach to classify infective keratitis. Curr. Eye Res..

[22] Loo J., Kriegel M.F., Tuohy M.M., Kim K.H., Prajna V., Woodward M.A., Farsiu S. (2020). Open-source automatic segmentation of ocular structures and biomarkers of microbial keratitis on slit-lamp photography images using deep learning. IEEE J. Biomed. Health Inf..

[23] Li D.-J., Huang B.-L., Peng Y. (2023). Comparisons of artificial intelligence algorithms in automatic segmentation for fungal keratitis diagnosis by anterior segment images. Front. Neurosci..

[24] Tiwari M., Piech C., Baitemirova M., Prajna N.V., Srinivasan M., Lalitha P., Villegas N., Balachandar N., Chua J.T., Redd T. (2022). Differentiation of Active Corneal Infections from Healed Scars Using Deep Learning. Ophthalmology.

[25] Kuo M.-T., Hsu B.W.-Y., Yin Y.-K., Fang P.-C., Lai H.-Y., Chen A., Yu M.-S., Tseng V.S. (2020). A deep learning approach in diagnosing fungal keratitis based on corneal photographs. Sci. Rep..

[26] Kuo M.-T., Hsu B.W.-Y., Lin Y.-S., Fang P.-C., Yu H.-J., Chen A., Yu M.-S., Tseng V.S. (2021). Comparisons of deep learning algorithms for diagnosing bacterial keratitis via external eye photographs. Sci. Rep..

[27] Wei Z., Wang S., Wang Z., Zhang Y., Chen K., Gong L., Li G., Zheng Q., Zhang Q., He Y. (2023). Development and multi-center validation of machine learning model for early detection of fungal keratitis. EBioMedicine.

[28] Natarajan R., Matai H.D., Raman S., Kumar S., Ravichandran S., Swaminathan S., Rani Alex J.S. (2022). Advances in the diagnosis of herpes simplex stromal necrotising keratitis: A feasibility study on deep learning approach. Indian J. Ophthalmol..

[29] Alquran H., Al-Issa Y., Alsalatie M., Mustafa W.A., Qasmieh I.A., Zyout A.a. (2022). Intelligent Diagnosis and Classification of Keratitis. Diagnostics.

[30] Hung N., Kang E.Y.-C., Shih A.G.-Y., Lin C.-H., Kuo M.T., Hwang Y.-S., Wu W.-C., Kuo C.-F., Hsiao C.-H. (2021). Using Slit-Lamp Images for Deep Learning–Based Identification of Bacterial and Fungal Keratitis. Diagnostics.

[31] Ghosh A.K., Thammasudjarit R., Jongkhajornpong P., Attia J., Thakkinstian A. (2022). Deep Learning for Discrimination Between Fungal Keratitis and Bacterial Keratitis: DeepKeratitis. Cornea.

[32] Won Y.K., Lee H., Kim Y., Han G., Chung T.-Y., Ro Y.M., Lim D.H. (2023). Deep learning-based classification system of bacterial keratitis and fungal keratitis using anterior segment images. Front. Med..

[33] Zhang Z., Wang H., Wang S., Wei Z., Zhang Y., Wang Z., Chen K., Ou Z., Liang Q. (2022). Deep learning-based classification of infectious keratitis on slit-lamp images. Ther. Adv. Chronic Dis..

[34] Kuo M.-T., Hsu B.W.-Y., Lin Y.S., Fang P.-C., Yu H.-J., Hsiao Y.-T., Tseng V.S. (2022). Deep Learning Approach in Image Diagnosis of Pseudomonas Keratitis. Diagnostics.

[35] Hu S., Sun Y., Li J., Xu P., Xu M., Zhou Y., Wang Y., Wang S., Ye J. (2023). Automatic Diagnosis of Infectious Keratitis Based on Slit Lamp Images Analysis. J. Pers. Med..

[36] Koyama A., Miyazaki D., Nakagawa Y., Ayatsuka Y., Miyake H., Ehara F., Sasaki S.-i., Shimizu Y., Inoue Y. (2021). Determination of probability of causative pathogen in infectious keratitis using deep learning algorithm of slit-lamp images. Sci. Rep..

[37] Sajeev S., Senthil M.P. Classifying infective keratitis using a deep learning approach. Proceedings of the 2021 Australasian Computer Science Week Multiconference.

[38] Wang L., Chen K., Wen H., Zheng Q., Chen Y., Pu J., Chen W. (2021). Feasibility assessment of infectious keratitis depicted on slit-lamp and smartphone photographs using deep learning. Int. J. Med. Inf..

[39] Redd T.K., Prajna N.V., Srinivasan M., Lalitha P., Krishnan T., Rajaraman R., Venugopal A., Acharya N., Seitzman G.D., Lietman T.M. (2022). Image-based differentiation of bacterial and fungal keratitis using deep convolutional neural networks. Ophthalmol. Sci..

[40] Xu Y., Kong M., Xie W., Duan R., Fang Z., Lin Y., Zhu Q., Tang S., Wu F., Yao Y.F. (2021). Deep sequential feature learning in clinical image classification of infectious keratitis. Engineering.

[41] Li Z., Jiang J., Chen K., Chen Q., Zheng Q., Liu X., Weng H., Wu S., Chen W. (2021). Preventing corneal blindness caused by keratitis using artificial intelligence. Nat. Commun..

[42] Kogachi K., Lalitha P., Prajna N.V., Gunasekaran R., Keenan J.D., Campbell J.P., Song X., Redd T.K. (2023). Deep Convolutional Neural Networks Detect no Morphological Differences Between Culture-Positive and Culture-Negative Infectious Keratitis Images. Transl. Vis. Sci. Technol..

[43] Tabatabaei S.A., Soleimani M., Tabatabaei S.M., Beheshtnejad A.H., Valipour N., Mahmoudi S. (2020). The use of in vivo confocal microscopy to track treatment success in fungal keratitis and to differentiate between Fusarium and Aspergillus keratitis. Int. Ophthalmol..

[44] Wu X., Qiu Q., Liu Z., Zhao Y., Zhang B., Zhang Y., Wu X., Ren J. (2018). Hyphae detection in fungal keratitis images with adaptive robust binary pattern. IEEE Access.

[45] Liu Z., Cao Y., Li Y., Xiao X., Qiu Q., Yang M., Zhao Y., Cui L. (2020). Automatic diagnosis of fungal keratitis using data augmentation and image fusion with deep convolutional neural network. Comput. Methods Programs Biomed..

[46] Hou H., Cao Y., Cui X., Liu Z., Xu H., Wang C., Zhang W., Zhang Y., Fang Y., Geng Y. (2021). Medical image management and analysis system based on web for fungal keratitis images. Math. Biosci. Eng..

[47] Zhang X., Ding G., Gao C., Li C., Hu B., Zhang C., Wang Q. Deep Learning for Three Types of Keratitis Classification based on Confocal Microscopy Images. Proceedings of the 2020 3rd International Conference on Signal Processing and Machine Learning.

[48] Tang N., Huang G., Lei D., Jiang L., Chen Q., He W., Tang F., Hong Y., Lv J., Qin Y. (2023). An artificial intelligence approach to classify pathogenic fungal genera of fungal keratitis using corneal confocal microscopy images. Int. Ophthalmol..

[49] Essalat M., Abolhosseini M., Le T.H., Moshtaghion S.M., Kanavi M.R. (2023). Interpretable deep learning for diagnosis of fungal and acanthamoeba keratitis using in vivo confocal microscopy images. Sci. Rep..

